# Sleep duration and psychotic experiences in patients at risk of psychosis: A secondary analysis of the EDIE-2 trial

**DOI:** 10.1016/j.schres.2018.08.006

**Published:** 2019-02

**Authors:** S. Reeve, A. Nickless, B. Sheaves, J. Hodgekins, S.L.K. Stewart, A. Gumley, D. Fowler, A. Morrison, D. Freeman

**Affiliations:** a Department of Psychiatry, University of Oxford, Warneford Hospital, Oxford, UK; b Nuffield Department of Primary Care Health Sciences, University of Oxford, Radcliffe Observatory Quarter, Woodstock Road, Oxford, UK; c Oxford Health NHS Foundation Trust, Oxford, UK; d Norwich Medical School, University of East Anglia, Norwich, UK; e Department of Psychology, University of Chester, Parkgate Road, Chester, UK; f Institute of Health and Wellbeing, University of Glasgow, Gartnavel Royal Hospital, Glasgow, UK; g School of Psychology, Pevensey Building, University of Sussex, Falmer, Brighton, UK; h Division of Psychology and Mental Health, University of Manchester, Manchester, UK

**Keywords:** At risk, Sleep, Psychosis, Longitudinal

## Abstract

Sleep disturbance is common among individuals at risk of psychosis, yet few studies have investigated the relationship between sleep disturbance and clinical trajectory. The Early Detection and Intervention Evaluation (EDIE-2) trial provides longitudinal data on sleep duration and individual psychotic experiences from a cohort of individuals at risk of psychosis, which this study utilises in an opportunistic secondary analysis. Shorter and more variable sleep was hypothesised to be associated with more severe psychotic experiences and lower psychological wellbeing. Mixed effect models were used to test sleep duration and range as predictors of individual psychotic experiences and psychological wellbeing over the 12–24 months (with assessments every 3 months) in 160 participants. Shorter sleep duration was associated with more severe delusional ideas and hallucinations cross-sectionally and longitudinally. The longitudinal relationships did not remain significant after conservative controls were added for the previous severity of psychotic experiences. No significant relationships were found between the sleep variables and other psychotic experiences (e.g. cognitive disorganisation), or psychological wellbeing. The results support a relationship between shorter sleep duration and delusional ideas and hallucinations. Future studies should focus on improving sleep disturbance measurement, and test whether treating sleep improves clinical trajectory in the at-risk group.

## 1 Introduction

A causal role for sleep disturbance in contributing to psychotic experiences has recently begun to emerge (Reeve et al., 2015). It has been demonstrated that reducing sleep increases psychotic experiences (Reeve et al., 2018), and that treating insomnia improves psychotic experiences (Freeman et al., 2017). Sleep disorders have also been shown to be common and severe, but treatable, among individuals with psychotic disorders (Freeman et al., 2009, Freeman et al., 2015). However there has been a surprising lack of research specifically investigating the causal contribution of sleep disturbance to psychotic experiences in at-risk individuals (Davies et al., 2016; Reeve et al., 2015; Zanini et al., 2013). This is despite a longstanding clinical awareness of sleep disturbance as a risk indicator for transition from the at-risk state to psychosis (Keshavan et al., 2004; Ruhrmann et al., 2010)

Existing research does indicate that sleep abnormalities are common in individuals at-risk of psychosis. Between 37% and 78% of this population have been found to report sleep disturbance, as assessed on the Scale of Assessment of Positive Symptoms (SAPS) across several studies (Miller et al., 2003; Poe et al., 2017). Reporting sleep disturbance has also been found to be highly predictive of transition to psychotic episode in the following 18 month period (Ruhrmann et al., 2010). However, ‘sleep disturbance’ is a broad term comprising a range of sleep disorders and disturbances (e.g. insomnia, circadian rhythm disruption, nightmares). This in turn hinders further research on how sleep disturbance in this group may contribute to psychotic experiences or transition to psychosis, or how it may be clinically addressed. In contrast, sleep recording studies have identified a range of specific sleep abnormalities in individuals at-risk of psychosis, including longer sleep onset latency, reduced sleep efficiency, and increased circadian rhythm disturbance in comparison to non-clinical controls (Lunsford-Avery et al., 2015; Zanini et al., 2015). These objective sleep disturbances have been linked with increased positive symptoms both at baseline and at 12 month follow up (Lunsford-Avery et al., 2015, Lunsford-Avery et al., 2016). In summary, research to date supports that sleep disturbance is common and relevant to clinical trajectory in psychosis, yet there needs to be further consensus on which sleep variables are relevant in this relationship. Especially valuable would be identification of specific self-reported sleep disturbances that are predictive of psychotic experiences, given the lack of access to objective sleep recording measures in standard clinical services.

One shortcoming across this literature has been the measurement of psychosis; in all the studies discussed above individual psychotic experiences were grouped together under the measurement of positive symptoms. However, multiple sources of evidence indicate that paranoia, hallucinations, grandiosity, and cognitive disorganisation exist as independent continua in the population with somewhat differing risk, development, and maintenance factors (Ronald, 2015; van Os, 2014; Zavos et al., 2014). Furthermore, as reported in a recent experimental study, sleep loss affects the dimensions of psychotic experience differently, with significant effects on hallucinations, paranoia, and cognitive disorganisation, but no effect on grandiosity (Reeve et al., 2018). Therefore, measuring individual psychotic experience dimensions may allow a clearer picture to emerge of the relationships between sleep disturbance and psychotic experiences in the at-risk population.

### 1.1 The Early Detection and Intervention Evaluation Trial (EDIE-2)

The EDIE-2 trial data may provide an opportunity to address some of these issues. EDIE-2 was a multi-site randomised controlled trial of a psychological intervention aiming to reduce transition to psychosis (Morrison et al., 2011, Morrison et al., 2012). A large cohort of individuals at risk for psychosis (*n* = 288) were recruited from five sites in the UK, and randomised such that half received a cognitive therapy based intervention and monitoring of mental state, and the other half received monitoring only. The intervention provided was based on a published treatment manual, and focused on psychotic experiences, although individual goals and formulations would have varied (French and Morrison, 2004). The cognitive therapy based intervention plus monitoring was significantly better than monitoring only in reducing severity of psychotic experiences, but not in reducing distress or transition to psychosis over 12 to 24 months (Morrison et al., 2012).

Most relevant for current purposes, one of the questionnaires assessed time spent asleep on ‘good’ and ‘bad’ nights over the previous three months. This provides a measure of sleep duration, alongside an indirect metric of the quality and variability of sleep by contrasting the ‘good’ and ‘bad’ duration – thereby allowing sleep reporting beyond endorsement of general sleep disturbance. As study assessments were carried out every three months for a minimum of 12 months (and up to 24 months), the EDIE-2 dataset has the most assessment time points, and longest follow up period, of any study recording sleep in the at-risk population to date. Furthermore, the assessment of psychotic experiences was carried out using the Comprehensive Assessment of At Risk Mental States (CAARMS), in which the subscales of the ‘positive psychotic experiences’ dimension allow measurement of individual psychotic experiences (Yung et al., 2003). These design characteristics support the use of this dataset for an opportunistic secondary analysis of the relationship between sleep and individual psychotic experiences over time in the at-risk group.

### 1.2 The current study

This study focuses on a secondary analysis of the EDIE-2 trial dataset with the aim of testing if sleep disturbance is significantly associated with more severe individual psychotic experiences cross-sectionally and longitudinally in a cohort of individuals at-risk of psychosis. A secondary aim was to test if sleep disturbance was cross-sectionally associated with depression or lower quality of life in this group.

Sleep disturbance in this study is operationalised by using the ‘bad night’ sleep duration and the difference (i.e. range) between the ‘bad night’ and ‘good night’ sleep duration, as recorded in the EDIE-2 study time use measure. The expectation is that a shorter bad night sleep duration and larger range of sleep duration suggest secondary aspects of sleep disturbance from sleep disorders. For example, insomnia would be associated with short sleep duration, and often high variability (as individuals oversleep to catch up on sleep lost on bad nights). Circadian rhythm disorders would also typically be associated with high variability, and possible short duration, as the preferred sleep window clashes with daily commitments.

## 2 Method

### 2.1 Participants

Two hundred and eighty-eight individuals aged 14–35 years participated in the EDIE-2 trial. Participants were assessed with the CAARMS to confirm they fulfilled the inclusion criterion of being at-risk of psychosis (intermittent psychotic symptoms, attenuated psychotic symptoms, or a decline in functioning alongside having a first degree relative with psychotic disorder). Exclusion criteria included current or previous receipt of antipsychotic medication, moderate to severe learning disability, organic impairment, and insufficient fluency in English. Half of the participants were assigned to cognitive therapy plus monitoring of mental state and the other half to monitoring of mental state only. All participants were followed up for a minimum of 12 months and a maximum of 24 months. For full details of trial design and results see Morrison et al., 2011, Morrison et al., 2012.

Sleep duration data were not retrievable from the Birmingham (*n* = 60) or Cambridge (*n* = 20) trial sites. Participants without a time point where both good sleep and bad sleep duration data had been reported were also excluded (*n* = 48). This resulted in a final sample of 160 individuals for the current study.

### 2.2 Measures

#### 2.2.1 Sleep

Self-reported sleep duration on ‘good’ and ‘bad’ nights over the past three months was collected within the Economic Patient Questionnaire interview (developed from the Time Use Survey; Short, 2006). Participants estimated their average sleep duration on ‘good’ and ‘bad’ nights over the last three months. The bad night sleep duration, and the range of sleep duration (i.e. the difference between the good and bad night sleep durations) were used in the current study. This information was collected at baseline, 3, 6, 9, 12, 15, 18, 21, and 24 months.

#### 2.2.2 Psychotic experiences

The CAARMS (Yung et al., 2003) was the primary measure of psychotic experiences in the EDIE-2 trial. The positive dimension was used in the current study, which comprises four psychotic experience subscales: Unusual Thought Content (UTC; e.g. ideas of reference, delusional mood, thought insertion), Non-Bizarre Ideas (NBI; e.g. persecutory ideas, grandiose ideas), Perceptual Abnormalities (PA; e.g. visual or auditory changes) and Disorganised Speech (DS; e.g. circumstantial or tangential speech).

Each subscale is rated by the interviewer for severity (0 to 6 scale, where 0 is not present and 6 is a clinical psychosis level), frequency and duration (0 to 6 scale, where 0 is absent, and 6 is continuous), and distress (0 to 100 scale, where 0 is not at all distressed, and 100 is extremely distressed). The severity and frequency are multiplied together to make a total severity score for the dimension. The individual distress scores are averaged to produce an overall distress score. Higher scores in all dimensions indicate greater severity, with scores ranging from 0 to 36 for the severity subscales and 0 to 100 for the distress subscale. CAARMS data from the baseline, 3, 6, 9, 12, 15, 18, 21, and 24 month assessments were used in the current study.

#### 2.2.3 Depression and quality of life

Depression was assessed using the Beck Depression Inventory for Primary Care (BDI-PC; Beck et al., 1997). This is a self-report questionnaire comprising 7 statements, rated on a 0 to 3 scale (where 0 is “Did not apply to me”, and 3 is “Applied to me very much or all the time”). The overall score is the sum of these items, of which the maximum score is 21. The BDI-PC was completed at baseline, 6 months, and 12 months.

The Manchester Short Assessment of Quality of Life (MANSA; Priebe et al., 1999) was used to measure satisfaction and quality of life. The measure comprises 12 items in which the participant assesses satisfaction in domains such as quality of friendships, financial situation, and physical health. Each item is rated on a 1 to 7 scale where 1 is a negative extreme (“Couldn't be worse”), and 7 is a positive extreme (“Couldn't be better”). The lowest possible score is 12, and the highest possible score is 84, with higher scores indicating increased satisfaction and higher quality of life. The MANSA was completed at baseline, 6 months and 12 months.

### 2.3 Analysis

All analyses were carried out using SPSS version 23 (IBM Corp., 2015). First, descriptive statistics were calculated for all study measures, and used to observe trends over the study. Differences between treatment allocation groups for each study measure (from all time points post-baseline) were then tested in independent samples *t*-tests.

**Fig. 1:**
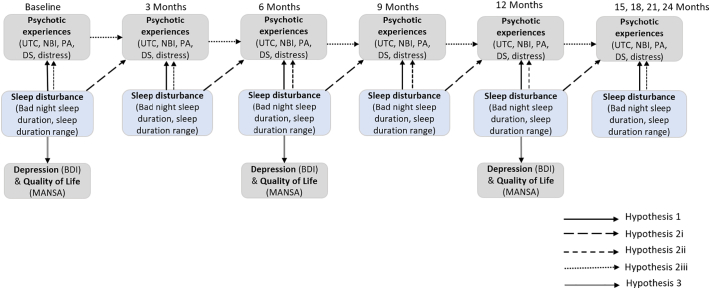
Analysis plan diagram.

The primary analysis approach was to run a set of pre-specified stepped linear mixed effects models to address each of the study hypotheses, as listed below. All models were tested even if no relationship was found at a prior phase. Linear mixed effects models allow examination of within subject correlations cross-sectionally and longitudinally by nesting the observations within individuals and time points, and allow use of all available data under the assumption that data are missing at random. The threshold for significance was set at *p* ≤ 0.05 for all analyses. Site and treatment allocation were included as factors in all models. A diagram of the analysis process can be found in Fig. 1.

#### 2.3.1 Hypothesis 1: Shorter ‘Bad night’ sleep duration and wider range in sleep duration will be associated with more severe psychotic experiences

Models were fitted to each dimension of the CAARMS (UTC, NBI, PA, DS, and Distress) with bad night sleep duration and sleep duration range as factors to test this cross-sectional hypothesis.

#### 2.3.2 Hypothesis 2: Previous shorter ‘Bad’ night sleep duration and wider range in sleep duration will predict later psychotic experience severity

This hypothesis was tested in several stages as below, with each stage acting as an increasingly conservative framing of the hypothesis via addition of control variables.

#### Step 1: previous sleep disturbance variables as standalone predictors

In this first step the longitudinal relationship between sleep and psychotic experiences was assessed by testing previous bad night sleep duration and sleep duration range as predictors of psychotic experiences at the next assessment time point in a linear mixed effects model.

#### Step 2: controlling for later sleep disturbance

In the second step the sleep variables at the later assessment time points were added to the model from Step 1. This allows a test of whether the previous sleep variables are significantly predictive of later psychotic experiences, once the expected relationship between sleep variables at the previous and later time points is controlled for.

#### Step 3: controlling for previous psychotic experience severity

In the final step previous psychotic experience severity was added to the model from Step 2. This tested if the coefficient of previous sleep disturbance continued to be significant when accounting for the expected relationship between previous and current psychotic experiences (as for the expected relationship between previous and current sleep as tested in Step 2).

#### 2.3.3 Hypothesis 3: Shorter ‘Bad night’ sleep duration and wider sleep duration range will be associated with increased depression and reduced quality of life

A cross-sectional linear mixed effects model (similar to Hypothesis 1) was fitted to depression (BDI-PC) and quality of life (MANSA) outcomes.

## 3 Results

### 3.1 Demographic and descriptive data

Demographic information for the study group can be found in Table 1. The group was predominantly male (*n* = 98, 61.1%), and the average age was 20.7 years. The majority were white (*n* = 147, 91.8%). The study group was balanced between interventions (*n* = 80, 50%, in each group).

**Table 1:** Demographics.

Age − mean (SD)	20.9 (4.2)
Gender − n (%)	
Male	98 (61.1)
Female	62 (38.9)
Ethnicity − n (%)	
White	147 (91.8)
Black (African/Caribbean/British/Other)	6 (3.8)
Asian (Indian/Pakistani/British/Chinese/Other)	3 (1.9)
Other	4 (2.5)
Randomisation − n (%)	
Monitoring + cognitive therapy	80 (50.0)
Monitoring only	80 (50.0)

Descriptive statistics for all the study variables can be found in Table 2. There was a general improvement in sleep over the study period: bad night sleep duration slightly increased, and the sleep duration range decreased. Bad night sleep duration was significantly negatively correlated with sleep duration range (*r* = −0.756, *p* < 0.001). There was also a general improvement in psychotic experience severity and distress across the study, and in depression and quality of life.

**Table 2:** Descriptive statistics for the study measures.

	Baseline		3 months		6 months		9 months		12 months		15 months		18 months		21 months		24 months	
	Mean (SD)	N	Mean (SD)	N	Mean (SD)	N	Mean (SD)	N	Mean (SD)	N	Mean (SD)	N	Mean (SD)	N	Mean (SD)	N	Mean (SD)	N
**Sleep duration (h)**																		
‘Good’ night	8.28 (2.5)	153	8.24 (2.2)	103	8.01 (2.2)	92	8.30 (2.4)	91	8.23 (2.2)	94	8.32 (2.3)	57	7.79 (2.5)	47	8.37 (2.0)	41	7.65 (1.6)	37
‘Bad’ night	4.14 (3.4)	136	4.54 (2.8)	96	4.81 (3.3)	90	4.58 (2.7)	88	4.45 (2.9)	91	5.27 (3.4)	52	4.94 (3.8)	40	5.49 (3.6)	40	4.99 (3.5)	36
Range	4.01 (3.6)	136	3.65 (3.1)	96	3.29 (3.1)	89	3.76 (3.4)	88	3.82 (3.3)	90	3.07 (3.4)	52	2.89 (4.5)	40	2.90 (3.5)	39	2.73 (3.0)	35

**CAARMS dimensions**																		
UTC	8.26 (7.7)	160	3.91 (6.5)	119	3.69 (6.0)	115	3.49 (6.4)	105	3.93 (6.9)	109	3.06 (5.7)	65	3.09 (6.6)	55	2.65 (4.8)	48	2.00 (4.5)	43
NBI	13.58 (7.1)	160	7.05 (7.4)	119	6.04 (6.8)	115	5.79 (6.9)	105	6.30 (8.0)	109	6.20 (6.9)	65	5.07 (6.7)	55	6.40 (7.4)	48	6.93 (9.0)	43
PA	10.11 (7.1)	160	5.49 (6.1)	119	4.72 (6.1)	115	4.79 (5.6)	106	4.90 (6.6)	108	4.23 (6.0)	65	4.25 (6.6)	55	4.54 (7.5)	48	5.16 (7.5)	43
DS	4.23 (4.8)	160	2.73 (4.1)	119	2.10 (3.6)	115	2.44 (4.0)	105	3.41 (4.8)	109	2.52 (4.1)	65	2.51 (3.4)	55	3.06 (4.7)	48	4.37 (6.9)	43
Distress	43.19 (20.8)	153	23.41 (22.1)	111	18.81 (20.9)	111	19.78 (20.4)	103	19.53 (17.9)	105	18.86 (21.3)	63	16.09 (19.2)	54	19.36 (22.6)	48	18.89 (21.3)	40

**Wellbeing**																		
MANSA	50.3 (10.7)	122			54.98 (11.7)	84			56.42 (13.3)	84								
BDI-PC	10.16 (4.4)	148			6.25 (4.6)	108			6.63 (5.2)	103								

CAARMS = Comprehensive Assessment of At Risk Mental States; UTC = Unusual Thought Content; NBI = Non-Bizarre Ideas; PA = Perceptual Abnormalities; DS = Disorganised Speech; BDI-PC – Beck Depression Inventory for Primary Care; MANSA = Manchester Short Assessment of Quality of Life.

There were several significant differences in outcomes between the treatment groups, as can be seen in Table 3. The cognitive therapy group had lower severity in the Unusual Thought Content and Perceptual Abnormality dimensions of the CAARMS than those who received monitoring alone. The group receiving cognitive therapy also had a significantly narrower range of sleep duration (*p* = 0.004), and showed a trend level significance for longer bad night sleep duration (*p* = 0.067). No other significant differences were found between the treatment groups.

**Table 3:** Comparison between treatment groups on sleep, psychotic experiences, and wellbeing (all time points except baseline).

	Group means (SD)		Independent samples t-test	
	Monitoring only	Monitoring and CBT	*t*-value	*p*-value
Sleep				
BadDur	4.53 (3.3)	5.03 (3.0)	−1.84	0.067
Range	3.82 (3.5)	2.97 (3.1)	2.93	0.004
CAARMS dimensions				
Distress	19.24 (20.0)	17.33 (19.0)	1.53	0.127
UTC	4.73 (7.5)	3.39 (5.9)	3.15	0.002
NBI	6.66 (7.9)	6.28 (7.5)	0.78	0.435
PA	5.13 (6.5)	4.06 (5.7)	2.82	0.005
DS	3.38 (5.0)	3.26 (4.8)	0.39	0.696
Wellbeing				
MANSA	58.28 (12.6)	56.34 (13.3)	1.19	0.237
BDI-PC	5.55 (4.8)	5.62 (4.9)	−0.14	0.889

BadDur = Bad night sleep duration (hours).

Range = Sleep duration range (hours).

Data included from all time points except baseline.

### 3.2 Relationship between sleep and psychotic experiences

#### 3.2.1 Hypothesis one: a cross-sectional model

The results from this analysis are summarised in Table 4. Shorter bad night sleep duration was significantly associated with increased severity of non-bizarre ideas (NBI), perceptual abnormalities (PA), and psychotic experience distress. Narrower range of sleep duration was significantly related to increased severity of NBI. No significant relationships between bad night sleep duration or sleep duration range were found for either unusual thought content (UTC) or disorganised speech (DS) in this cross-sectional model.

**Table 4:** Mixed effect model output – hypothesis one (cross-sectional only).

	Factor	Estimate	Std. Error	df	*t*	*p*	95% confidence interval	
							Lower bound	Upper bound
Unusual thought content	BadDur	−0.059	0.11	541.9	−0.52	0.602	−0.28	0.16
	Range	−0.010	0.11	583.8	−0.09	0.926	−0.23	0.21
Non-bizarre ideas	BadDur	−0.402	0.14	610.3	−2.88	0.004	−0.68	−0.13
	Range	−0.331	0.13	578.0	−2.53	0.012	−0.59	−0.07
Perceptual abnormalities	BadDur	−0.327	0.13	615.7	−2.59	0.010	−0.57	−0.08
	Range	0.003	0.12	600.2	0.02	0.983	−0.23	0.24
Disorganised speech	BadDur	−0.041	0.08	612.5	−0.53	0.596	−0.19	0.11
	Range	0.026	0.07	586.5	0.36	0.719	−0.12	0.17
Distress	BadDur	−1.170	0.42	589.8	−2.79	0.005	−1.99	−0.35
	Range	−0.415	0.39	566.1	−1.07	0.287	−1.18	0.35

BadDur = Bad night sleep duration (hours).

Range = Sleep duration range (hours).

Treatment allocation and site controlled for in all analyses.

In summary, a shorter bad night sleep duration was cross-sectionally associated with increased delusional ideas and hallucinations (and distress) as hypothesised. However, narrower sleep range was significantly related to increased severity of delusional ideas, contrary to our hypothesis.

#### 3.2.2 Hypothesis two: Longitudinal models

##### 3.2.2.1 Step 1: a longitudinal model

The results from this analysis are summarised in Table 5. The longitudinal relationships were similar to those in the cross-sectional model in that there were significant relationships between previous shorter bad night sleep duration and increased severity of later NBI and PA, although the relationship with distress did not reach significance when tested longitudinally (*p* = 0.078). Smaller sleep duration range at the previous time point was significantly related to increased severity of later NBI. No significant relationships were found between the previous sleep variables and later UTC or DS.

**Table 5:** Mixed effect model output – hypothesis two (step one -without current sleep control).

	Factor	Estimate	Std. Error	df	*t*	*p*	95% Confidence Interval	
							Lower	Upper
Psychotic Experiences (CAARMS)								
Unusual thought content	t-1_BadDur	−0.167	0.13	464.5	−1.31	0.190	−0.42	0.08
	t-1_Range	−0.154	0.12	446.4	−1.28	0.202	−0.39	0.08
Non-bizarre ideas	t-1_BadDur	−0.432	0.15	440.7	−2.96	0.003	−0.72	−0.14
	t-1_Range	−0.281	0.14	393.7	−2.07	0.039	−0.55	−0.01
Perceptual abnormalities	t-1_BadDur	−0.324	0.14	467.5	−2.39	0.017	−0.59	−0.06
	t-1_Range	0.009	0.13	458.6	0.07	0.947	−0.24	0.26
Disorganised speech	t-1_BadDur	−0.116	0.09	465.9	−1.35	0.178	−0.29	0.05
	t-1_Range	0.005	0.08	437.3	0.06	0.954	−0.15	0.16
Distress	t-1_BadDur	−0.812	0.46	469.8	−1.77	0.078	−1.71	0.09
	t-1_Range	−0.369	0.43	444.2	−0.86	0.389	−1.21	0.47

BadDur = Bad night sleep duration (hours).

Range = Sleep duration range (hours).

t-1 = from previous time point.

Treatment allocation and site controlled for in all analyses.

These results suggest that shorter bad night sleep duration is a significant predictor of later delusional ideas and hallucinations. Narrower sleep range was associated with increased severity of later delusional ideas, again contrary to hypothesis.

##### 3.2.2.2 Step 2: a longitudinal model controlling for later sleep

The results from this analysis can be found in Table 6. Previous short bad night sleep duration remained a significant predictor for later NBI, even once sleep at the later time point was controlled for. However, neither bad sleep duration nor sleep duration range (at either the previous or later time point) had a significant predictive effect on other psychotic experience severities.

**Table 6:** Mixed effect model output – hypothesis two (step two - with current sleep control).

	Factor	Estimate	Std. Error	df	*t*	*p*	95% Confidence Interval	
							Lower	Upper
Unusual Thought Content	BadDur	−0.052	0.15	327.9	−0.34	0.736	−0.36	0.25
	Range	0.005	0.14	395.2	0.04	0.971	−0.27	0.28
	t-1_BadDur	−0.132	0.15	341.1	−0.89	0.374	−0.42	0.16
	t-1_Range	−0.106	0.14	381.0	−0.77	0.443	−0.38	0.17
Non-Bizarre Ideas	BadDur	−0.384	0.18	368.9	−2.10	0.036	−0.74	−0.02
	Range	−0.401	0.16	374.1	−2.51	0.012	−0.71	−0.09
	t-1_BadDur	−0.383	0.18	390.6	−2.18	0.030	−0.73	−0.04
	t-1_Range	−0.193	0.16	372.8	−1.23	0.220	−0.50	0.12
Perceptual Abnormalities	BadDur	−0.184	0.17	399.6	−1.06	0.291	−0.53	0.16
	Range	0.077	0.15	402.0	0.51	0.613	−0.22	0.38
	t-1_BadDur	−0.259	0.17	409.4	−1.56	0.121	−0.59	0.07
	t-1_Range	0.018	0.15	397.0	0.12	0.902	−0.28	0.31
Disorganised Speech	BadDur	−0.052	0.11	402.8	−0.47	0.637	−0.27	0.16
	Range	0.002	0.09	369.8	0.02	0.986	−0.18	0.19
	t-1_BadDur	−0.030	0.11	414.4	−0.29	0.775	−0.24	0.18
	t-1_Range	0.062	0.09	379.8	0.67	0.503	−0.12	0.25
Distress	BadDur	−1.080	0.58	386.5	−1.87	0.062	−2.22	0.05
	Range	−0.409	0.49	373.4	−0.83	0.406	−1.37	0.56
	t-1_BadDur	−0.146	0.54	393.9	−0.27	0.788	−1.22	0.92
	t-1_Range	0.101	0.48	354.2	0.21	0.833	−0.84	1.04

BadDur = Bad night sleep duration (hours).

Range = Sleep duration range (hours).

t-1 = from previous time point.

Treatment allocation and site controlled for in all analyses.

These findings suggest that the relationship between previous sleep disturbance and later hallucinations may be accounted for previous sleep disturbance predicting later sleep disturbance. However, previous bad night sleep duration was still a significant additional predictor of later delusional ideas, even once sleep disturbance at the later time point was accounted for.

##### 3.2.2.3 Step 3: a longitudinal model controlling for previous psychotic experience severity

Table 7 shows the results from this phase of analysis. In all cases the previous severity of each psychotic experience is a significant positive predictor of later severity of that psychotic experience. The only sleep relationship remaining significant in these final models is a cross-sectional relationship between shorter bad night sleep and more severe NBI. No previous sleep disturbance variables remained significant predictors once previous psychotic experiences were accounted for.

**Table 7:** Mixed effect model output – hypothesis two (step three – with controls for current sleep and previous psychotic experience).

	Factor	Estimate	Std. Error	df	*t*	*p*	95% confidence interval	
							Lower	Upper
Unusual Thought Content	BadDur	−0.121	0.14	332.3	−0.87	0.385	−0.39	0.15
	Range	0.009	0.12	351.0	0.07	0.944	−0.23	0.25
	t-1_BadDur	0.009	0.13	342.4	0.06	0.949	−0.25	0.27
	t-1_Range	−0.031	0.12	357.1	−0.26	0.798	−0.27	0.21
	t-1_UTC	0.395	0.04	370.5	10.23	0.000	0.32	0.47
Non-Bizarre Ideas	BadDur	−0.365	0.16	387.3	−2.28	0.023	−0.68	−0.05
	Range	−0.226	0.14	369.3	−1.64	0.101	−0.50	0.04
	t-1_BadDur	−0.036	0.15	397.9	−0.23	0.816	−0.34	0.27
	t-1_Range	−0.059	0.13	355.2	−0.43	0.664	−0.32	0.21
	t-1_NBI	0.477	0.04	389.5	12.59	0.000	0.40	0.55
Perceptual Abnormalities	BadDur	−0.181	0.16	402.4	−1.14	0.256	−0.49	0.13
	Range	0.070	0.14	386.9	0.51	0.614	−0.20	0.34
	t-1_BadDur	−0.082	0.15	404.3	−0.54	0.589	−0.38	0.22
	t-1_Range	−0.032	0.14	386.2	−0.23	0.815	−0.30	0.23
	t-1_PA	0.406	0.04	401.4	9.88	0.000	0.33	0.49
Disorganised Speech	BadDur	−0.051	0.11	399.6	−0.48	0.629	−0.26	0.16
	Range	−0.013	0.09	354.9	−0.15	0.883	−0.19	0.17
	t-1_BadDur	−0.011	0.10	413.0	−0.11	0.911	−0.21	0.19
	t-1_Range	0.034	0.09	375.7	0.38	0.703	−0.14	0.21
	t-1_DS	0.286	0.05	381.5	6.25	0.000	0.20	0.38
Distress	BadDur	−1.158	0.60	371.5	−1.92	0.056	−2.35	0.03
	Range	−0.424	0.50	349.7	−0.84	0.400	−1.41	0.57
	t-1_BadDur	−0.037	0.57	377.3	−0.07	0.948	−1.15	1.08
	t-1_Range	0.194	0.49	332.1	0.40	0.691	−0.76	1.15
	t-1_Distress	0.032	0.01	83.4	2.36	0.020	0.01	0.06

BadDur = Bad night sleep duration (hours).

Range = Sleep duration range (hours).

t-1 = from previous time point.

Treatment allocation and site controlled for in all analyses.

This final set of models therefore suggest that previous psychotic experience is a stronger predictor than previous sleep disturbance for later psychotic experiences, with no evidence of a unique contribution of sleep disturbance across time for predicting change in psychotic experiences.

#### 3.2.3 Hypothesis three: sleep and psychological wellbeing

The outcome of this stage of analysis can be seen in Table 8. Neither bad night sleep duration, nor sleep duration range, were significantly associated with depression or quality of life in these cross-sectional models.

**Table 8:** Mixed effect model output – hypothesis three (cross-sectional, depression and quality of life).

	Factor	Estimate	Std. Error	df	t	*p*	95% confidence interval	
							Lower bound	Upper bound
Depression (BDI-PC)	BadDur	−0.161	0.13	288.5	−1.21	0.226	−0.42	0.10
	Range	−0.013	0.13	289.4	−0.10	0.920	−0.26	0.24
Quality of Life (MANSA)	BadDur	0.332	0.34	224.3	0.98	0.330	−0.34	1.00
	Range	0.084	0.34	231.0	0.25	0.806	−0.59	0.76

BadDur = Bad night sleep duration (h).

Range = Sleep duration range (h).

Treatment allocation and site controlled for in all analyses.

BDI-PC – Beck Depression Inventory for Primary Care; MANSA = Manchester Short Assessment of Quality of Life.

## 4 Discussion

The aim of this study was to utilise a large dataset to perform an opportunistic investigation of the relationship between sleep duration and psychotic experiences in patients at high risk of psychosis. As hypothesised, shorter sleep duration on bad nights was cross-sectionally associated with significantly more delusional ideas and hallucinations, and with higher levels of distress from psychotic experiences. However, a smaller sleep duration range was associated with more severe delusional ideas, contrary to the hypothesised positive association. Similar relationships were present longitudinally, with shorter bad night sleep duration at the previous time point predicting later severity of hallucinations and delusional ideas, as hypothesised. However, when additional controls were added these relationships did not remain significant. Overall the results support further investigation in to the relationship between sleep disturbance and individual psychotic experiences.

The significant associations between reduced bad night sleep duration and increased delusional ideas and hallucinations in the present study are consistent with the links between reduced sleep and increased paranoia and hallucinations as reported in a previous experimental study (Reeve et al., 2018). The association between narrower range and increased psychotic experiences was contrary to the study hypothesis, and appears inconsistent with previous studies that have reported a link between increased sleep variability and psychotic experiences (Lunsford-Avery et al., 2016; Waters et al., 2011). However, in the context of the bad night sleep duration results the most consistent interpretation is that overall low sleep duration (i.e. short sleep on good and bad nights) is associated with more severe psychotic experiences.

These findings are consistent with theoretical models for sleep disruption and psychosis in the at-risk group. In one model sleep/wake disturbances are seen as both reflecting an underlying biological vulnerability and as an additional stressor. In other words, sleep disturbances are thought to initiate a vicious cycle wherein disrupted sleep leads to increased psychotic experiences, the distress from which disrupts sleep further (Lunsford-Avery and Mittal, 2013). It is also worth noting that participants who received the cognitive therapy intervention showed more improvement in sleep over the study period (as well as significantly less severe psychotic experiences). This supports a parallel between improving psychotic experiences and improved sleep, which could be investigated more directly with a sleep intervention study. A recent pilot case series with an at-risk population indicated that treating sleep problems did result in decreases in psychotic experiences and negative affect (Bradley et al., 2018). Sleep treatment therefore represents an exciting new possibility for at-risk intervention.

No significant relationships were found between the sleep variables and the psychotic experience dimensions of unusual thought content or disorganised speech, which indicates that the relationship between sleep disturbance and psychotic experiences may be somewhat selective, as has been reported elsewhere (Reeve et al., 2018). The findings from this study further support the importance of measuring individual psychotic experiences separately when investigating the influence of sleep.

The non-significant relationship between depression and either measure of sleep disturbance in the results is surprising. This is contrary to previous findings indicating negative affect as a mediator in the sleep disturbance and psychosis relationship (e.g. Reeve et al., 2018), and contrary to a general literature linking sleep disturbance with depression (Murphy and Peterson, 2015). The BDI-PC scale used in the current study is typically used as a screening tool with a categorical outcome (with scores ≥4 indicating major depression). Notably, the majority of the participants were above this cut-off throughout the trial. Nevertheless, as these scores were used dimensionally, this measure may have lacked sensitivity to detect changes. The null relationship between sleep and quality of life is also unexpected. Sleep problems reduce quality of life (Kyle et al., 2010a, Kyle et al., 2010b), and sleep and quality of life have both been linked to psychotic symptoms in clinical groups (Afonso et al., 2011). A potential explanation could be that fatigue is more linked than sleep to functional health (Waters et al., 2013), however as there was no measure of fatigue in the current study it was not possible to assess this. The null finding in both depression and quality of life could also be related to lower power in these tests, given the lower rate of completion of these measures.

One worthwhile issue to investigate is whether disrupted sleep is associated with transition to psychosis. This was not possible to test in this study as there was a very low transition rate to psychosis in the group (9.4%), lower than expected in a group of individuals fulfilling the at-risk criteria (typically >16%; Hartmann et al., 2016; Yung et al., 2008). This transition rate could have been lower due to a positive effect of the study monitoring on improving psychotic experiences. Indeed, as can be seen in the descriptive measures both study groups showed improvements in severity of psychotic experiences and other measures over time.

### 4.1 Limitations

Similar to previous large studies from the at-risk literature (Miller et al., 2003; Poe et al., 2017; Ruhrmann et al., 2010), the EDIE-2 study did not have sleep as a focus. However, one advantage of the current study is that the sleep information is not a single sleep quality judgement as in the SAPS, but gives duration information. It is also worth noting that youth at risk of psychosis have elsewhere been found to be accurate self-reporters of sleep duration (Lunsford-Avery et al., 2015). Secondly, in this study sleep information was collected at up to nine time points over two years. As a result, this goes beyond previous secondary analysis studies where sleep information was only collected at a maximum of two time points.

The sleep duration estimates have some limitations as measures of sleep disturbance. For example, the frequency of good nights versus bad nights was not assessed, which would have allowed calculation of an average sleep time and variability of sleep. The duration variable also does not give context on the timing of sleep, which is important as circadian dysfunction is commonly reported in individuals with psychosis (Wulff et al., 2012), and has been found to be predictive of positive symptoms in individuals at risk of psychosis (Lunsford-Avery et al., 2016).

One limitation in the statistical approach is co-linearity among the predictors. The two sleep variables were negatively correlated, such that individuals with shorter bad sleep duration had a wider sleep duration range (as would be expected within a common insomnia presentation). Sleep variables were also highly correlated over time. This decreases the possibility that individual variables will emerge as predictors of variance in the outcome (i.e. psychotic experience severity). One way to reduce this effect is to mean centre the variables within each participant. However, in the present study differences between the starting position for each participant is non-arbitrary (e.g. a change from 8 h to 6 h sleep on a bad night is unlikely to have the same effect as a change from 6 h to 4 h), therefore this option was not appropriate. Another way to remove this issue is to induce changes in the predictor variable across time (e.g. by providing an intervention), as in the pilot trial recently reported by Bradley et al. (2018).

A separate statistical limitation in this study is the need to control for site and treatment allocation, which will have reduced the power available to detect other effects in the remaining variance. This may have contributed to the low significance of the relationships reported, which in many cases would not survive conservative corrections for multiple testing. However, given that the approach here is of planned comparisons, where all analyses are pre-specified, it is not necessarily appropriate to apply these corrections (Feise, 2002; Schulz and Grimes, 2005). Furthermore, it was not possible to control for age or occupational status, which are likely to be a source of significant variance in sleep as reported in the current study. Substance abuse was also not accounted for in the current study.

Overall the findings show that sleep disturbance, even when indirectly measured, acts as a significant longitudinal predictor of later psychotic experiences in the at-risk population (up until the most conservative corrections are made). Prediction of clinical trajectories is particularly valuable for the at-risk group, therefore a possible role of sleep disturbance in predicting later outcomes deserves further investigation. This study also supports the potential importance of treating sleep disturbance in this group, a possibility now beginning to be explored in the clinical literature (Bradley et al., 2018). Future studies should investigate the effectiveness of these interventions on psychotic symptoms, and assess sleep disorder symptoms in clinical groups in order to refine intervention targets.

## Conflict of interest

BS provides clinical consultancy to Sleepio (Big Health Ltd.). No other conflicts of interest exist in relation to the subject of the study.

## Role of the funding source

None of the funders had a role in the design of the study, collection or analysis of data, or in preparation or submission of the manuscript.

## Contribution

SR, BS, DFr conceived the study and planned the analyses. AN was statistical consult for the analysis. SS was the trial manager on the EDIE2 trial. AG, DFo, and AM provided access to the data from the trial. All authors contributed to and have approved the final version of the manuscript.
